# A Supramolecular Extension of Mosher’s Method: Absolute Configuration Assignment of *N*-Amino Acid Derivatives via Bis-Thiourea Chiral Solvating Agent

**DOI:** 10.3390/molecules30142930

**Published:** 2025-07-11

**Authors:** Virginia Rondinini, Federica Aiello, Federica Cefalì, Alessandra Recchimurzo, Gloria Uccello Barretta, Federica Balzano

**Affiliations:** 1 Department of Chemistry and Industrial Chemistry, University of Pisa, Via G. Moruzzi 13, 56124 Pisa, Italy; virginia.rondinini@studio.unibo.it (V.R.); cefalifederica@gmail.com (F.C.); alessandra.recchimurzo06@gmail.com (A.R.); federica.balzano@unipi.it (F.B.); 2 National Research Council-Institute for Chemical and Physical Processes (CNR-IPCF), Via G. Moruzzi 1, 56124 Pisa, Italy

**Keywords:** CSA, NMR, absolute configuration, chiral analysis, ROESY, association constant, Mosher’s method

## Abstract

The bis-thiourea chiral solvating agent (CSA) **BTDA** enables the NMR-based determination of absolute configuration in *N*-3,5-dinitrobenzoyl (DNB) amino acid derivatives without requiring covalent derivatization. A reliable trend of the sense of nonequivalence and absolute configuration is found in both ^1^H and ^13^C NMR spectra. A dual-enantiomer approach, using (*R*,*R*)- and (*S*,*S*)-**BTDA**, generates diastereomeric complexes with the enantiopure substrate, and distinct spatial arrangements are reflected in consistent and interpretable Δδ values. The observed chemical shift differences correlate reliably with the stereochemistry of the chiral center and are further supported by ROESY (Rotating-frame Overhauser Enhancement SpectroscopY) experiments and binding constants’ measurements, confirming the formation of stereoselective non-covalent complexes. This methodology extends the logic of Mosher’s analysis to solvating agents and remains effective even in samples containing single pure enantiomers of the amino acid derivative. The **BTDA**-based dual-CSA system thus represents a robust, non-derivatizing strategy for stereochemical assignment by NMR, combining operational simplicity with broad applicability to DNB derivatives of amino acids with free carboxyl function.

## 1. Introduction

Amino acids are fundamental to biological systems, serving as building blocks of peptides and proteins. However, their stereochemical integrity can be compromised by racemization processes that occur under varying pH conditions [1], potentially altering the conformation and biological activity of supramolecular derivatives [2,3]. The accurate determination of stereoisomeric purity and absolute configuration is therefore essential, both in biological studies and in the development of stereoselective synthetic procedures [4,5,6,7,8,9].

Traditionally, circular dichroism (CD) and CD coupled with high-performance liquid chromatography–mass spectrometry have been used for configurational analysis and sequencing peptides [10,11,12,13,14,15]; chromatographic and electrophoretic techniques have been proved to be suitable for detecting chiral impurities at trace levels [16,17,18].

In the field more closely related to the determination of the enantiomeric composition and absolute configuration of chiral compounds, chromatographic Marfey’s method [19,20], based on the ultraviolet detection of amino acids derivatized with *N*-α-(2,4-dinitro-5-fluorophenyl)-l-alaninamide, remains widely used but it is sensitive to the enantiomeric purity of the derivatizing agent, which can impact the accuracy of enantiomeric composition analysis [19,20].

Analogously, chiral derivatizing agents (CDAs) are widely employed for the chiral analysis of several compounds by nuclear magnetic resonance (NMR) spectroscopy. Importantly, this technique allows for the analysis of any NMR-active nucleus present in the derivatized chiral substrate (e.g., ^1^H, ^13^C, ^31^P, ^19^F, ^77^Se, ^125^Te) [21,22,23,24,25,26,27].

In contrast, chiral solvating agents (CSAs) for NMR spectroscopy offer a non-covalent alternative. These form diastereomeric solvates with enantiomers via hydrogen bonding, dipole-dipole, or π-π interactions, enabling differentiation directly in NMR spectra. The relative positions of NMR signals (i.e., the sense of nonequivalence) correlate with absolute configuration, and the CSA’s enantiomeric purity affects only the extent of enantiomer differentiation, not the accuracy of enantiomeric composition. Chiral oriented media, instead, proved to be complementary efficient tools for the chiral discrimination of substrates lacking polar functional groups [28,29].

CSAs with rigid pre-organized structures can enhance enantiomeric discrimination through cooperative aromatic interactions and anisotropic effects. Thermodynamic control over solvation equilibria can be achieved by tuning concentration, molar ratio, and temperature [30,31,32].

Despite their potential, CSAs have been applied to a lesser extent for configuration assignment. Their application in the determination of the absolute configuration is mainly based on the observation of a reliable and reproducible trend in the sense of nonequivalence (the difference in chemical shifts of a selected nucleus of the substrate in each diastereomer) for a large number of analyzed substrates, i.e., on the trend in the relative position of (*R*)- and (*S*)-substrate in their corresponding diastereomers. Among those, a cationic cobalt(III) complex has been employed for the configurational analysis of fluorine-labeled amine and alcohol derivatives by ^19^F NMR [33]. ^31^P NMR approach to the configurational analysis of inositol phosphates has been reported by using l-arginine amide as CSA [34]. Novel tetraaza macrocyclic CSAs have been proposed for the chiral analysis, enantiomeric composition, and absolute configuration determination of thiohydantoin derivatives by ^1^H NMR spectroscopy [35].

A peculiar approach not based on chemical shift parameters has been recently reported by Cabral et al. [36] for small molecules by using a mixture of CSAs and comparing diffusion coefficients of diastereomeric aggregates determined by NMR.

A different approach has been outlined by using both enantiomers of the CSA in the analysis of a single enantiomer of chiral compounds, as in the case of benzamides based on *trans*-1,2-diaminocyclohexane fragment that have been applied for the absolute configuration determination of mandelic acid derivatives [37].

**Figure 1:**
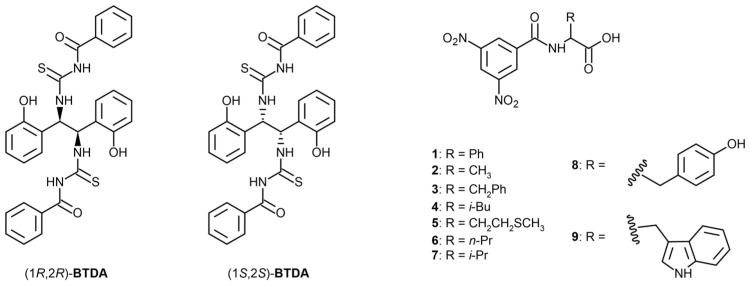
Structures of CSAs (1*R*,2*R*)- and (1*S*,2*S*)-**BTDA**, and of amino acid derivatives **1**–**9**.

CSAs have been rarely applied to amino acids and related derivatives. Recently, the ^1^H and ^19^F NMR chemical shift–absolute configuration correlation has been proven to be reliable for absolute configuration determination of *N*-trifluoracetyl amino acids in the presence of squaramide-based supramolecular CSA [38] or of quinine, a natural alkaloid-CSA, in a multicomponent mixture of amino acid derivatives [39]. The same approach allowed for the configurational analysis of *N*-acetyl amino acids by ^1^H NMR by using bis-arylthiourea CSA obtained starting from amino derivatives of isohexide and 1,4-diazabicyclo[2.2.2]octane (DABCO) as the third achiral component [40]. Notably, Song et al. demonstrated effective configurational assignment using both enantiomers of a bis-thiourea CSA for phthaloyl amino acids [41]. In 2020, our group introduced a thiourea-based CSA for *N*-3,5-dinitrobenzoyl (DNB) amino acids, yielding significant ^1^H and ^13^C differentiation [42] in the presence of DABCO. We later reported the synthesis of the bis-thiourea derivative (1*R*,2*R*)-**BTDA** (Figure 1), starting from (1*R*,2*R*)-1,2-bis(2-hydroxyphenyl)ethylenediamine ((1*R*,2*R*)-**DA**) [43], achieving strong enantiodiscrimination in similar experimental conditions on the same class of substrates.

Here, **BTDA** finds additional application in the determination of the absolute configuration of the same *N*-DNB amino acids (Figure 1) via ^1^H and ^13^C NMR spectroscopy. Moreover, the availability of both enantiomers of the diamine, (1*R*,2*R*)- and (1*S*,2*S*)-**DA**, enabled the preparation of (*R*,*R*)- and (*S*,*S*)-**BTDA**, allowing for a protocol conceptually akin to Mosher’s method [44], but based on supramolecular rather than covalent interactions. To validate this approach, ^1^H chemical-shift differences (Δδ^(*R*,*R*)−(*S*,*S*)^ = Δδ^(*R*,*R*)^ − Δδ^(*S*,*S*)^) and intermolecular dipolar interactions were measured, and association constants were calculated.

## 2. Results and Discussion

**Scheme 1:**
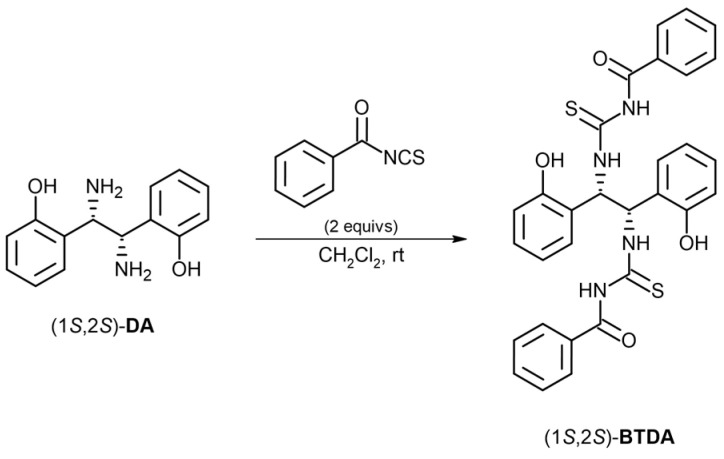
Synthesis of (1*S*,2*S*)-**BTDA**.

(1*S*,2*S*)-**BTDA** was synthetized according to the procedure already used for the synthesis of its enantiomer (1*R*,2*R*)-**BTDA** [43] (Scheme 1).

Four sets of ternary mixtures CSA/DABCO/*N*-DNB amino acid were prepared, containing either (*R*,*R*)- or (*S*,*S*)-**BTDA** (30 mM) and either one equivalent of (*R*)- or (*S*)-amino acid derivative. For each mixture, the proton (^1^H) NMR spectrum was recorded in deuterated chloroform (CDCl_3_) in the presence of one equivalent of DABCO, and the chemical shifts of selected resonances (the *ortho* and *para* protons of the dinitrobenzoyl moiety, and the methine proton of the chiral center) of the amino acid derivative were measured. By calculating the difference between the chemical shifts measured in the presence of (*R*,*R*)-**BTDA** and of (*S*,*S*)-**BTDA**, the parameter Δδ^(*R*,*R*)-(*S*,*S*)^ (Δδ^(*R*,*R*)^ − Δδ^(*S*,*S*)^) was determined in each mixture. In the case of substrates **8** and **9**, characterized by a lower solubility compared to substrates **1**–**7**, the concentration was lowered to 15 mM, and a second equivalent of DABCO was added. For these samples, therefore, the molar ratio CSA/DABCO/amino acid derivative was equal to 1:2:1.

**Figure 2:**
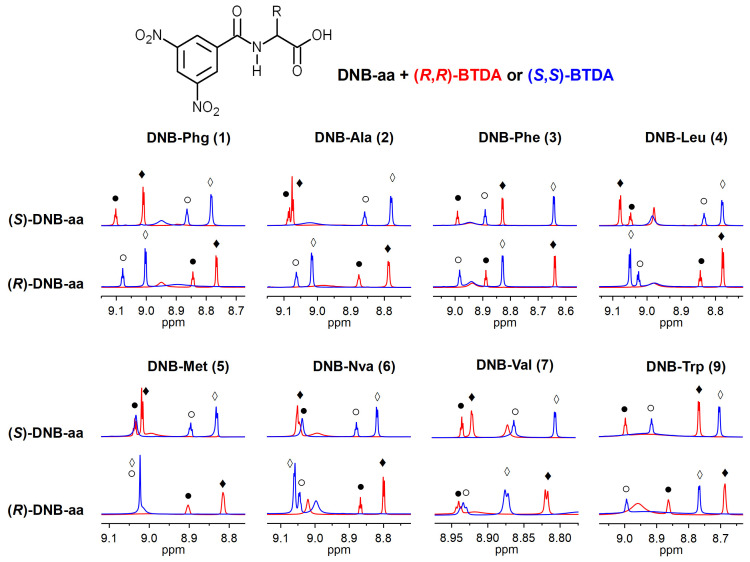
^1^H NMR (600 MHz, CDCl_3_, 298 K) spectral regions corresponding to *ortho* (diamond) and *para* (circle) protons of the DNB moiety of enantiopure **1**–***7*** (30 mM) and **9** (15 mM) in the presence of (*R*,*R*)-**BTDA** (red spectra and full symbols) or (*S*,*S*)-**BTDA** (blue spectra and empty symbols), and DABCO. Signals without symbols belong to the CSA.

The values of Δδ^(*R*,*R*)-(*S*,*S*)^ calculated for the two enantiomers of substrates **1**–**9** are collected in Table 1, whereas Figure 2 and Figure S1 (Supplementary Materials) show the spectral regions of *ortho*-DNB (*o*-DNB), *para*–DNB (*p*-DNB) and chiral methine protons in the four mixtures.

With the aim to extend the analytical application of **BDTA**, the possibility to use this CSA for the determination of the absolute configuration of enantiomerically enriched samples of *N*-DNB amino acids was evaluated. (*S*)-Enriched solutions of substrates **1**–**7** (30 mM) were prepared with one equivalent of (*R*,*R*)-**BTDA** and one equivalent of DABCO. (*R*)-Enriched substrates **8**–**9** (15 mM) were mixed with one equivalent of (*R*,*R*)-**BTDA** and two equivalents of DABCO.

**Figure 3:**
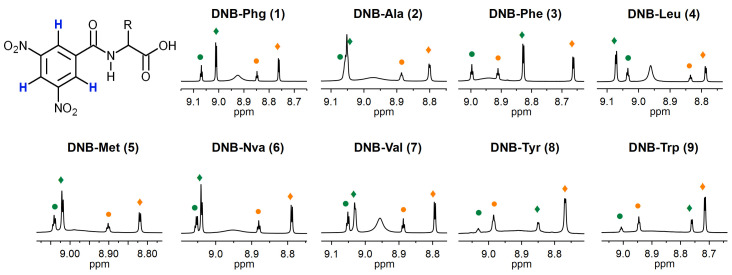
^1^H NMR (600 MHz, CDCl_3_, 298 K) spectral regions corresponding to *ortho* (diamond) and *para* (circle) protons of the DNB moiety of (*S*)-enantiomerically enriched samples of **1**–**7** (30 mM) in the presence of 1 equivalent of (*R*,*R*)-**BTDA** and DABCO and of (*R*)-enantiomerically enriched samples of **8** and **9** (15 mM) in the presence of 1 equivalent of (*R*,*R*)-BTDA and 2 equivalents of DABCO. Green symbols refer to the (*S*)-enantiomer, orange symbols refer to the (*R*)-enantiomer.

As reported in Figure 3, for all the substrates, the resonances of the *ortho*- and *para*-DNB protons of the (*S*)-enantiomer were shifted at higher frequency than those belonging to the (*R*)-enantiomer; according to what we already observed (Table 1 and Figure 2), an opposite sense of nonequivalence was found for the methine protons (Figure S2, Supplementary Materials).

**Figure 4:**
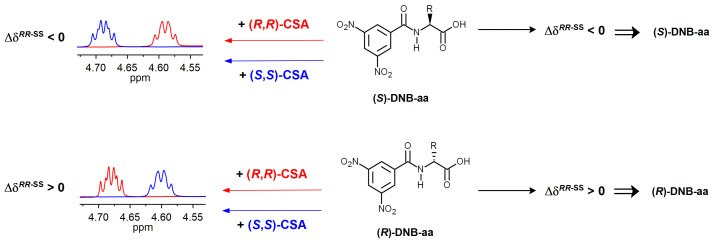
A schematic representation of the proposed methodology.

A schematic overview illustrating the method is reported in Figure 4 based on α-methine proton behavior.

Considering the promising results already obtained by exploiting ^13^C NMR analysis for configurational assignment [45], the correlation between the sense of nonequivalence and the absolute configuration was also evaluated by analyzing the chemical shifts of the quaternary carbons, namely the aromatic one bound to the nitro group and the amidic carbon. For substrates **8** and **9**, given the lower concentration of the solutions, the carbon spectra were not recorded.

**Figure 5:**
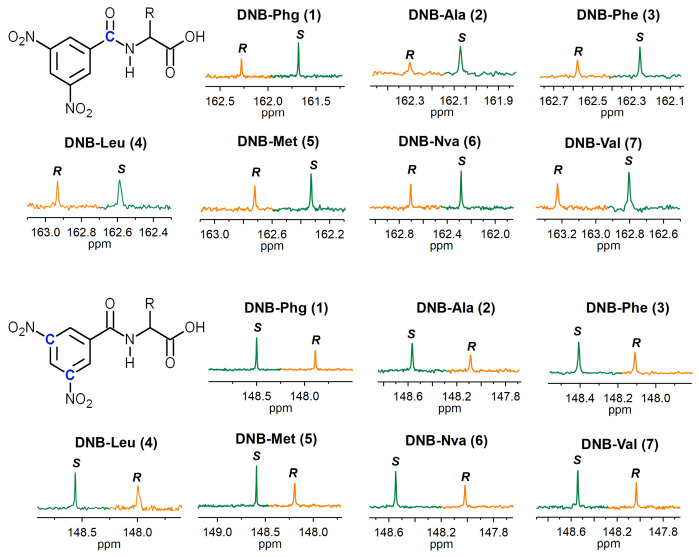
^13^C NMR (150 MHz, CDCl_3_, 298 K) spectral regions corresponding to the amidic (**top**) and C-NO_2_ (**bottom**) carbons of (*S*)-enantiomerically enriched samples of **1**–**7** (30 mM) in the presence of 1 equivalent of (*R*,*R*)-**BTDA** and DABCO.

A higher shift for the amidic carbon of the (*R*)-enantiomer was observed for all the diastereomeric derivatives formed with (*R*,*R*)-**BTDA**, while an opposite trend was found for the aromatic carbon, where the resonance belonging to the (*S*)-enantiomer was shifted at a higher frequency (Figure 5).

Interestingly, the data collected from the ^13^C NMR spectra showed a strong correlation with the results obtained from the analysis of the ^1^H NMR spectra. In fact, the sense of nonequivalence observed for the C-NO_2_ resonances was consistent to what was found for the *ortho*- and *para*-DNB protons (negative values of Δδ^(*R*,*R*)−(*S*,*S*)^); similarly, the methine proton and the amidic carbon were characterized by the same trend (positive values of Δδ^(*R*,*R*)−(*S*,*S*)^).

Aiming to obtain more insights into the discrimination processes, the association constant was determined for each enantiomer of the substrates **2**–**7** in mixture with DABCO and (*R*,*R*)-**BTDA**. A 1:1:1 complexation stoichiometry was assumed, and a nonlinear least-square fitting based on dilution data was carried out [46]. The association constants were not determined for substrates **8** and **9** due to their low solubility. *K_R_* and *K_S_* were calculated by using Equation (1):

(1) $$\delta_{obs}=\delta_{f}+\left(\delta_{b}-\delta_{f}\right)\frac{\sqrt{1+4C_{0}K}-1}{\sqrt{1+4C_{0}K}+1}$$

where *δ_obs_* is the chemical shift measured in the mixture with the CSA, *δ_b_* and *δ_f_* are the chemical shifts in the bound and the free state, respectively, *C*_0_ is the molar concentration of the substrate, and *K* is the association constant. By applying Equation (1), the constants reported in Table 2 were calculated (see also Figure S3, Supplementary Materials).

For all the substrates, the value calculated for *K_R_* was higher than *K_S_*, suggesting that the solvates formed by the (*R*)-enantiomer of each substrate with (*R*,*R*)-**BTDA** are more stable than those with the (*S)*-enantiomer.

Finally, 1D ROESY (Rotating-frame Overhauser Enhancement SpectroscopY) experiments were carried out for the four diastereomeric mixtures (*R*)-substrate/DABCO/(*R*,*R*)-**BTDA**, (*S*)-substrate/DABCO/(*R*,*R*)-**BTDA**, (*R*)-substrate/DABCO/(*S*,*S*)-**BTDA** and (*S*)-substrate/DABCO/(*S*,*S*)-**BTDA**. No significant differences were observed in the nature of intermolecular dipolar interactions among the different mixtures (Figures S4–S15, Supplementary Materials).

In all cases, inter-ROEs were detected between the 3,5-dinitrophenyl protons of the amino acid derivative and selected benzoyl and 2-hydroxyphenyl protons of the CSA (Figures S4, S7, S10 and S13, Supplementary Materials), as well as inter-ROEs between its methine proton and protons of the 2-hydroxyphenyl moiety (Figures S5, S8, S11 and S14, Supplementary Materials). These interactions only differed in their intensities, which were stronger for the (*R*,*R*)-CSA/(*R*)-substrate, and, specularly, (*S*,*S*)-CSA/(*S*)-substrate combinations, compared to the (*R*,*R*)-CSA/(*S*)-substrate and (*S*,*S*)-CSA/(*R*)-substrate ones.

Therefore, the interaction mechanism underlying the chiral discrimination exerted by **BTDA** in the presence of DABCO can be rationalized by considering the spatial complementarity between the CSA and the *N*-DNB amino acid derivatives.

DABCO acts as a proton shuttle (as confirmed by the intense ROEs produced on protons of the substrate and the CSA, Figures S6, S9, S12 and S15, Supplementary Materials), mediating hydrogen bond interactions between the carboxyl and amide functions of the substrate and the thiourea moieties and hydroxyl function of **BTDA**, thus stabilizing the formation of 1:1:1 supramolecular complexes.

**Figure 6:**
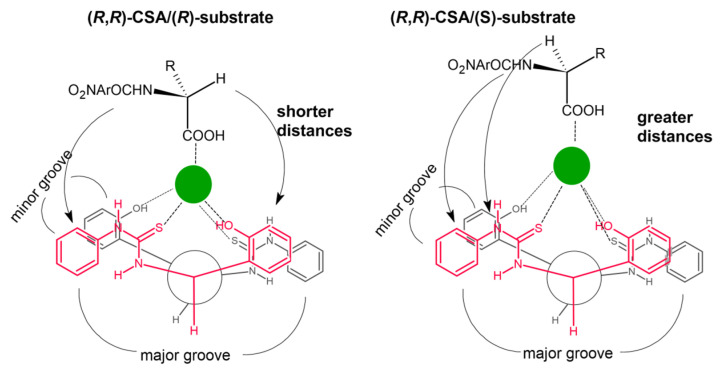
A representation of the interaction mechanism between (*R*,*R*)-**BTDA**, the two enantiomers of the substrates, and DABCO (green sphere).

The **BTDA** scaffold creates a preorganized chiral cavity [43] featuring two distinct binding regions, which may be described as a major groove and a minor groove (Figure 6). In the case of the (*R*)-enantiomer/(*R*,*R*)-CSA pair, the substrate preferentially interacts with the major groove formed by the 2-hydroxyphenyl and benzoyl moieties belonging to the same thiourea unit. This arrangement places the DNB ring of the substrate in close proximity to the benzoyl group, resulting in significant shielding of the *ortho*- and *para*-DNB protons, while the methine proton lies near the phenolic ring, experiencing a deshielding effect. The proximity of these interacting groups accounts for both the larger Δδ values observed in the ^1^H and ^13^C NMR spectra and the stronger ROE cross-peaks detected for the (*R*)-enantiomer. Moreover, this configuration leads to higher association constants, indicating a more stable complex formation. Conversely, the (*S*)-enantiomer interacts preferentially through the minor groove engaging the 2-hydroxyphenyl of one thiourea unit and the benzoyl group of the opposite arm. This less favorable geometry results in increased distances between the substrate’s DNB and methine protons and the corresponding aromatic surfaces of **BTDA**, leading to weaker anisotropic effects, smaller complexation shifts (Table S1, Supplementary Materials) and lower association constants.

The enantiodiscrimination thus emerges from the combined effect of bidentate hydrogen bonding, sterically guides accommodation, and different anisotropic shielding, producing consistent trends across the observed NMR parameters.

Beyond *N*-DNB amino acid derivatives, other *N*-protected substates (BOC, FMOC, CBZ), already tested for enantiodifferentiation with **BTDA** [43], could be used as substrates to extend its applicability in absolute configuration assignment. However, phenomena like equilibration (slow-exchange in NMR) between *syn*- and *anti*-stereoisomeric forms could take place [43] and have to be considered when analyzing the NMR data.

## 3. Materials and Methods

### 3.1. Materials

All the syntheses of sensitive compounds were carried out under dry nitrogen. All commercially available reagents, substrates, and solvents were purchased from Sigma Aldrich (Darmstadt, Germany). Deuterated chloroform (CDCl_3_) used in the NMR experiments was acquired from Deutero GmbH (Kastellaun, Germany).

### 3.2. Methods

^1^H and ^13^C{^1^H} NMR measurements were carried out in CDCl_3_ solution on a Varian INOVA spectrometer equipped with a 5 mm probe operating at 600 MHz and 150 MHz for ^1^H and ^13^C nuclei, respectively. ^1^H and ^13^C chemical shifts were referred to tetramethyl silane (TMS) as the secondary reference standard and the temperature was controlled (298 ± 0.1 K). The ^1^H NMR spectra were recorded by using the minimum spectral width required, a relaxion delay of 5 s, 1.8 s of acquisition time, and a gain of 20, and 32 scans; the π/2 pulse was optimized for each sample. The ^13^C{^1^H} NMR spectra were recorded using a relaxion delay of 1 s, 0.9 s of acquisition time, a gain of 30, and 14,400 scans. The 1D-ROESY spectra were recorded using a selective inversion pulse with 512 scans, a relaxion delay of 5 s, and a mixing time of 0.5 s.

### 3.3. Synthesis of (S,S)-BTDA

The bis-thiourea was synthetized according to the literature [43]. NMR characterization data for both the CSA and the DNB amino acids are available in the literature [43,45].

### 3.4. Sample Preparation for the NMR Analysis

Chiral solvating agents (*R*,*R*)- or (*S*,*S*)-**BTDA** (30 mM with **1**–**7**, 15 mM with **8** and **9**), DABCO (30 mM), and (*R*)-/(*S*)-DNB amino acids (30 mM for **1**–**7**, 15 mM for **8** and **9**) were mixed in CDCl_3_ (0.6 mL) to reach a substrate/CSA/DABCO molar ratio of 1:1:1 for substrates **1**–**7** (30 mM) and 1:1:2 for substrates **8** and **9**.

Enantiomerically enriched samples were prepared by mixing the proper amount of (*R*)-enantiomer and (*S*)-enantiomer for each substrate, then (*R*,*R*)-**BTDA** and DABCO were added to reach a substrate/CSA/DABCO molar ratio of 1:1:1 for substrates **1**–**7** (30 mM) and 1:1:2 for substrates **8** and **9**.

Dilution experiments for determining the association constants were performed by preparing a stock solution of substrate/(*R*,*R*)-**BTDA**/DABCO and by diluting it properly to reach the following concentrations: 50 mM, 45 mM, 40 mM, 35 mM, 30 mM, 25 mM, 20 mM, 15 mM, 10 mM, 5 mM, 2.5 mM, 1 mM, 0.5 mM.

## 4. Conclusions

Despite the central role of the amino acids in biological, pharmaceutical, and biomedical fields, nowadays there are less NMR methods for the differentiation of their enantiomers, and especially for the assignment of their absolute configuration.

The results presented in this study confirm the stereodiscriminating capability of **BTDA** as a bis-thiourea chiral solvating agent for the non-derivatizing NMR-based assignment of absolute configuration in *N*-DNB amino acid derivatives. The use of both enantiomers of **BTDA** enables a dual-CSA protocol that yields reproducible and interpretable Δδ patterns in NMR spectra. These differences correlate unambiguously with the amino acid derivative stereochemistry, even when working with single enantiomers.

The method circumvents the need for covalent modification of the substrate, offering a reliable and operationally simple alternative to classical derivatization-based strategies. Experimental support from ROESY and association constant data further substantiates the formation of distinct, non-covalent diastereomeric complexes.

The stereoselectivity observed in the **BTDA**-based system can be rationalized considering the spatial complementarity between the CSA’s pre-organized bis-thiourea scaffold and the *N*-DNB amino acid derivatives. The dual thiourea moieties provide a bidentate hydrogen-bonding framework, while the flanking aromatic units of **BTDA** enable π-π stacking with the electron-deficient dinitrophenyl group. These interactions are further stabilized by the presence of DABCO, which may serve as a spatial orienting cofactor, bridging between the carboxyl function of the amino acid derivatives and the CSA scaffold. The distinct Δδ trends observed in the ^1^H and ^13^C NMR spectra are consistent with a preferential fit of each enantiomer into a specific CSA chirality, driven by cumulative hydrogen bonding and aromatic interactions, and the differences in association constants support this stereochemically guided binding model.

Beyond the specific case of **BTDA**, this study highlights the broader potential of dual-CSA approach in NMR spectroscopy as a general platform for stereochemical analysis. Future developments may extend this strategy to other classes of chiral compounds, fostering the design of tailored solvating agents with enhanced selectivity and broader chemical compatibility. The method has a general applicability to any kind of CSA, provided that the CSA has stereoelectronic features suitable for differentiating the enantiomers of the analyzed substrates and the two enantiomers of the CSA are available.

## Figures and Tables

**Table 1:** Δδ^(*R*,*R*)-(*S*,*S*)^ (ppm) values calculated from the ^1^H NMR (600 MHz, CDCl_3_, 298 K) spectra of **1**–**7** (30 mM) and of **9** (15 mM), in the presence of 1 equivalent of (*R*,*R*)- or (*S*,*S*)-**BTDA**, and 1 equivalent (**1**–**7**) or 2 equivalents of DABCO (**9**) ^1^.

Substrate	*p*-DNB	*o*-DNB	CH-α	Substrate	*p*-DNB	*o*-DNB	CH-α
(*R*)-**1**	−0.234	−0.236	0.071	(*S*)-**1**	0.238	0.227	−0.097
(*R*)-**2**	−0.186	−0.292	0.115	(*S*)-**2**	0.212	0.275	−0.107
(*R*)-**3**	−0.095	−0.189	0.052	(*S*)-**3**	0.101	0.186	−0.053
(*R*)-**4**	−0.181	−0.271	0.076	(*S*)-**4**	0.215	0.299	−0.032
(*R*)-**5**	−0.118	−0.206	0.043	(*S*)-**5**	0.138	0.184	−0.090
(*R*)-**6**	−0.177	−0.261	0.080	(*S*)-**6**	0.165	0.236	−0.097
(*R*)-**7**	−0.192	−0.117	0.080	(*S*)-**7**	0.241	0.150	−0.072
(*R*)-**9**	−0.132	−0.080	0.130	(*S*)-**9**	0.082	0.064	−0.070

^1^ No data for substrate **8** are reported due to unavailability of both enantiopure derivatives.

**Table 2:** Association constants (*K*, M^−1^) calculated on the *p*-DNB proton of the (*R*)- and (*S*)-enantiomer of **1**–**7** (30 mM) for equimolar diastereomeric mixtures containing DABCO and (*R*,*R*)-**BTDA**.

Substrate	*K* *_R_*	*K* *_S_*
**1**	297 ± 13 ^1^	54 ± 3 ^1^
**2**	161 ± 6	72 ± 11
**3**	114 ± 3	35 ± 1
**4**	266 ± 19	10 ± 2
**5**	100 ± 8	58 ± 6
**6**	137 ± 7	7 ± 1
**7**	227 ± 5	58 ± 6

^1^ data reported in ref. [43].

## Data Availability

Data are available upon request.

## Supplementary Materials

The following supporting information can be downloaded at: https://www.mdpi.com/article/10.3390/molecules30142930/s1, Figure S1: ^1^H NMR (600 MHz, CDCl_3_, 298 K) spectral regions corresponding to chiral methine proton of enantiopure **1**–**7** (30 mM) and **9** (15 mM) in the presence of (*R*,*R*)-**BTDA** (red spectra and full symbols) or (*S*,*S*)-**BTDA** (blue spectra and empty symbols), and DABCO; Figure S2: ^1^H NMR (600 MHz, CDCl_3_, 298 K) spectral regions corresponding to the methine proton of (*S*)-enantiomerically enriched samples of **1**–**7** (30 mM) in the presence of 1 equivalent of (*R*,*R*)-**BTDA** and DABCO and of (*R*)-enantiomerically enriched samples of **8** and **9** (15 mM) in the presence of 1 equivalent of (*R*,*R*)-**BTDA** and 2 equivalents of DABCO. Green symbols refer to the (*S*)-enantiomer, orange symbols refer to the (*R*)-enantiomer; Figure S3: Association constants calculated for the two enantiomers of substrates **2**–**7** in mixture with (*R*,*R*)-**BTDA** and DABCO; Figure S4: 1D-ROESY (600 MHz, CDCl_3_, 298 K, mixing time 500 ms) spectra of *ortho*-protons of **2** in (a) (*R*,*R*)-**BTDA**/(*R*)-**2**, (b) (*S*,*S*)-**BTDA**/(*R*)-**2**, (c) (*R*,*R*)-**BTDA**/(*S*)-**2**, (d) (*S*,*S*)-**BTDA**/(*S*)-**2**; Figure S5: 1D-ROESY (600 MHz, CDCl_3_, 298 K, mixing time 500 ms) spectra of chiral methine proton of **2** in (a) (*R*,*R*)-**BTDA**/(*R*)-**2**, (b) (*S*,*S*)-**BTDA**/(*R*)-**2**, (c) (*R*,*R*)-**BTDA**/(*S*)-**2**, (d) (*S*,*S*)-**BTDA**/(*S*)-**2**; Figure S6: 1D-ROESY (600 MHz, CDCl_3_, 298 K, mixing time 500 ms) spectra of methylene protons of DABCO in (a) (*R*,*R*)-**BTDA**/(*R*)-**2**, (b) (*S*,*S*)-**BTDA**/(*R*)-**2**, (c) (*R*,*R*)-**BTDA**/(*S*)-**2**, (d) (*S*,*S*)-**BTDA**/(*S*)-**2**. ▼ refers to CSA proton signals, * refers to substrate proton signals; Figure S7: 1D-ROESY (600 MHz, CDCl_3_, 298 K, mixing time 500 ms) spectra of *ortho*-protons of **3** in (a) (*R*,*R*)-**BTDA**/(*R*)-**3**, (b) (*S*,*S*)-**BTDA**/(*R*)-**3**, (c) (*R*,*R*)-**BTDA**/(*S*)-**3**, (d) (*S*,*S*)-**BTDA**/(*S*)-**3**. Signals without symbols belong to the substrate; Figure S8: 1D-ROESY (600 MHz, CDCl_3_, 298 K, mixing time 500 ms) spectra of chiral methine proton of **3** in (a) (*R*,*R*)-**BTDA**/(*R*)-**3**, (b) (*S*,*S*)-**BTDA**/(*R*)-**3**, (c) (*R*,*R*)-**BTDA**/(*S*)-**3**, (d) (*S*,*S*)-**BTDA**/(*S*)-**3**. Signals without symbols belong to the substrate; Figure S9: 1D-ROESY (600 MHz, CDCl_3_, 298 K, mixing time 500 ms) spectra of methylene protons of DABCO in (a) (*R*,*R*)-**BTDA**/(*R*)-**3**, (b) (*S*,*S*)-**BTDA**/(*R*)-**3**, (c) (*R*,*R*)-**BTDA**/(*S*)-**3**, (d) (*S*,*S*)-**BTDA**/(*S*)-**3**. ▼ refers to CSA proton signals, * refers to substrate proton signals; Figure S10: 1D-ROESY (600 MHz, CDCl_3_, 298 K, mixing time 500 ms) spectra of *ortho*-protons of **4** in (a) (*R*,*R*)-**BTDA**/(*R*)-**4**, (b) (*S*,*S*)-**BTDA**/(*R*)-**4**, (c) (*R*,*R*)-**BTDA**/(*S*)-**4**, (d) (*S*,*S*)-**BTDA**/(*S*)-**4**; Figure S11: 1D-ROESY (600 MHz, CDCl_3_, 298 K, mixing time 500 ms) spectra of chiral methine proton of **4** in (a) (*R*,*R*)-**BTDA**/(*R*)-**4**, (b) (*S*,*S*)-**BTDA**/(*R*)-**4**, (c) (*R*,*R*)-**BTDA**/(*S*)-**4**, (d) (*S*,*S*)-**BTDA**/(*S*)-**4**; Figure S12: 1D-ROESY (600 MHz, CDCl_3_, 298 K, mixing time 500 ms) spectra of methylene protons of DABCO in (a) (*R*,*R*)-**BTDA**/(*R*)-**4**, (b) (*S*,*S*)-**BTDA**/(*R*)-**4**, (c) (*R*,*R*)-**BTDA**/(*S*)-**4**, (d) (*S*,*S*)-**BTDA**/(*S*)-**4**. ▼ refers to CSA proton signals, * refers to substrate proton signals; Figure S13: 1D-ROESY (600 MHz, CDCl_3_, 298 K, mixing time 500 ms) spectra of *ortho*-protons of **5** in (a) (*R*,*R*)-**BTDA**/(*R*)-**5**, (b) (*S*,*S*)-**BTDA**/(*R*)-**5**, (c) (*R*,*R*)-**BTDA**/(*S*)-**5**, (d) (*S*,*S*)-**BTDA**/(*S*)-**5**; Figure S14: 1D-ROESY (600 MHz, CDCl_3_, 298 K, mixing time 500 ms) spectra of chiral methine proton of **5** in (a) (*R*,*R*)-**BTDA**/(*R*)-**5**, (b) (*S*,*S*)-**BTDA**/(*R*)-**5**, (c) (*R*,*R*)-**BTDA**/(*S*)-**5**, (d) (*S*,*S*)-**BTDA**/(*S*)-**5**; Figure S15: 1D-ROESY (600 MHz, CDCl_3_, 298 K, mixing time 500 ms) spectra of methylene protons of DABCO in (a) (*R*,*R*)-**BTDA**/(*R*)-**5**, (b) (*S*,*S*)-**BTDA**/(*R*)-**5**, (c) (*R*,*R*)-**BTDA**/(*S*)-**5**, (d) (*S*,*S*)-**BTDA**/(*S*)-**5**. ▼ refers to CSA proton signals, * refers to substrate proton signals; Table S1: Complexation shift (Δδ = δ_obs_ − δ_free_, ppm) of *para*- and *ortho*-proton of DNB moiety and of α-CH proton of both enantiomers of **1**–**7** (30 mM), **8** and **9** (15 mM) in the presence of 1 equivalent of (*R*,*R*)-**BTDA**, 1 equivalent (**1**–**7**) or 2 equivalents of DABCO (**8**,**9**).
