# Supplementary material for: A Supramolecular Extension of Mosher’s Method: Absolute Configuration Assignment of N-Amino Acid Derivatives via Bis-Thiourea Chiral Solvating Agent
Source: Molecules. 2025 Jul 11;30(14):2930. doi: 10.3390/molecules30142930 (PMC12298456; doi:10.3390/molecules30142930)
Supplement: Supplementary file 1 [file molecules-30-02930-s001.zip › molecules-3740893-supplementary.pdf]

A Supramolecular Extension of Mosher's Method: Absolute Configuration Assignment of *N*-Amino Acid Derivatives via Bis-Thiourea Chiral Solvating Agent

Virginia Rondinini [1], Federica Aiello [2,*], Federica Cefalì [1], Alessandra Recchimurzo [1], Gloria Uccello Barretta [1,2,*] and Federica Balzano [1]

1 Department of Chemistry and Industrial Chemistry, University of Pisa, Via G. Moruzzi 13, 56124 Pisa, Italy; virginia.rondinini@studio.unibo.it (V.R.); cefalifederica@gmail.com (F.C.); alessandra.recchimurzo06@gmail.com (A.R.); federica.balzano@unipi.it (F.B.)

2 National Research Council-Institute for Chemical and Physical Processes (CNR-IPCF), Via G. Moruzzi 1, 56124 Pisa, Italy

* Correspondence: federica.aiello@cnr.it (F.A.); gloria.uccello.barretta@unipi.it (G.U.B.)

Supplementary Materials

Table of contents

Figure S1. ^{1}H NMR (600 MHz, $CDCl_3$, 298 K) spectral regions corresponding to chiral methine proton of enantiopure **1**-**7** (30 mM) and **9** (15 mM) in the presence of (*R*,*R*)-**BTDA** (red spectra and full symbols) or (*S*,*S*)-**BTDA** (blue spectra and empty symbols), and DABCO.

Figure S2. ^{1}H NMR (600 MHz, $CDCl_3$, 298 K) spectral regions corresponding to the methine proton of (*S*)-enantiomerically enriched samples of **1**-**7** (30 mM) in the presence of 1 equiv of (*R*,*R*)-**BTDA** and DABCO and of (*R*)-enantiomerically enriched samples of **8** and **9** (15 mM) in the presence of 1 equiv of (*R*,*R*)-BTDA and 2 equivs of DABCO. Green symbols refer to the (*S*)-enantiomer, orange symbols refer to the (*R*)-enantiomer.

Figure S3. Association constants calculated for the two enantiomers of substrates **2**-**7** in mixture with (*R*,*R*)-**BTDA** and DABCO.

Figure S4. 1D-ROESY (600 MHz, $CDCl_3$, 298 K, mixing time 500 ms) spectra of *ortho*-protons of **2** in (a) (*R*,*R*)-**BTDA**/(*R*)-**2**, (b) (*S*,*S*)-**BTDA**/(*R*)-**2**, (c) (*R*,*R*)-**BTDA**/(*S*)-**2**, (d) (*S*,*S*)-**BTDA**/(*S*)-**2**.

Figure S5. 1D-ROESY (600 MHz, $CDCl_3$, 298 K, mixing time 500 ms) spectra of chiral methine proton of **2** in (a) (*R*,*R*)-**BTDA**/(*R*)-**2**, (b) (*S*,*S*)-**BTDA**/(*R*)-**2**, (c) (*R*,*R*)-**BTDA**/(*S*)-**2**, (d) (*S*,*S*)-**BTDA**/(*S*)-**2**.

Figure S6. 1D-ROESY (600 MHz, $CDCl_3$, 298 K, mixing time 500 ms) spectra of methylene protons of DABCO in (a) (*R*,*R*)-**BTDA**/(*R*)-**2**, (b) (*S*,*S*)-**BTDA**/(*R*)-**2**, (c) (*R*,*R*)-**BTDA**/(*S*)-**2**, (d) (*S*,*S*)-**BTDA**/(*S*)-**2**. ▼ refers to CSA proton signals, * refers to substrate proton signals.

Figure S7. 1D-ROESY (600 MHz, $CDCl_3$, 298 K, mixing time 500 ms) spectra of *ortho*-protons of **3** in (a) (*R*,*R*)-**BTDA**/(*R*)-**3**, (b) (*S*,*S*)-**BTDA**/(*R*)-**3**, (c) (*R*,*R*)-**BTDA**/(*S*)-**3**, (d) (*S*,*S*)-**BTDA**/(*S*)-**3**. Signals without symbols belong to the substrate.

Figure S8. 1D-ROESY (600 MHz, $CDCl_3$, 298 K, mixing time 500 ms) spectra of chiral methine proton of **3** in (a) (*R*,*R*)-**BTDA**/(*R*)-**3**, (b) (*S*,*S*)-**BTDA**/(*R*)-**3**, (c) (*R*,*R*)-**BTDA**/(*S*)-**3**, (d) (*S*,*S*)-**BTDA**/(*S*)-**3**. Signals without symbols belong to the substrate.

Figure S9. 1D-ROESY (600 MHz, $CDCl_3$, 298 K, mixing time 500 ms) spectra of methylene protons of DABCO in (a) (*R*,*R*)-**BTDA**/(*R*)-**3**, (b) (*S*,*S*)-**BTDA**/(*R*)-**3**, (c) (*R*,*R*)-**BTDA**/(*S*)-**3**, (d) (*S*,*S*)-**BTDA**/(*S*)-**3**. ▼ refers to CSA proton signals, * refers to substrate proton signals.

Figure S10. 1D-ROESY (600 MHz, $CDCl_3$, 298 K, mixing time 500 ms) spectra of *ortho*-protons of **4** in (a) (*R*,*R*)-**BTDA**/(*R*)-**4**, (b) (*S*,*S*)-**BTDA**/(*R*)-**4**, (c) (*R*,*R*)-**BTDA**/(*S*)-**4**, (d) (*S*,*S*)-**BTDA**/(*S*)-**4**.

Figure S11. 1D-ROESY (600 MHz, $CDCl_3$, 298 K, mixing time 500 ms) spectra of chiral methine proton of **4** in (a) (*R*,*R*)-**BTDA**/(*R*)-**4**, (b) (*S*,*S*)-**BTDA**/(*R*)-**4**, (c) (*R*,*R*)-**BTDA**/(*S*)-**4**, (d) (*S*,*S*)-**BTDA**/(*S*)-**4**.

Figure S12. 1D-ROESY (600 MHz, $CDCl_3$, 298 K, mixing time 500 ms) spectra of methylene protons of DABCO in (a) (*R*,*R*)-**BTDA**/(*R*)-**4**, (b) (*S*,*S*)-**BTDA**/(*R*)-**4**, (c) (*R*,*R*)-**BTDA**/(*S*)-**4**, (d) (*S*,*S*)-**BTDA**/(*S*)-**4**. ▼ refers to CSA proton signals, * refers to substrate proton signals.

Figure S13. 1D-ROESY (600 MHz, $CDCl_3$, 298 K, mixing time 500 ms) spectra of *ortho*-protons of **5** in (a) (*R*,*R*)-**BTDA**/(*R*)-**5**, (b) (*S*,*S*)-**BTDA**/(*R*)-**5**, (c) (*R*,*R*)-**BTDA**/(*S*)-**5**, (d) (*S*,*S*)-**BTDA**/(*S*)-**5**.

Figure S14. 1D-ROESY (600 MHz, $CDCl_3$, 298 K, mixing time 500 ms) spectra of chiral methine proton of **5** in (a) (*R*,*R*)-**BTDA**/(*R*)-**5**, (b) (*S*,*S*)-**BTDA**/(*R*)-**5**, (c) (*R*,*R*)-**BTDA**/(*S*)-**5**, (d) (*S*,*S*)-**BTDA**/(*S*)-**5**.

Figure S15. 1D-ROESY (600 MHz, $CDCl_3$, 298 K, mixing time 500 ms) spectra of methylene protons of DABCO in (a) (*R*,*R*)-**BTDA**/(*R*)-**5**, (b) (*S*,*S*)-**BTDA**/(*R*)-**5**, (c) (*R*,*R*)-**BTDA**/(*S*)-**5**, (d) (*S*,*S*)-**BTDA**/(*S*)-**5**. ▼ refers to CSA proton signals, * refers to substrate proton signals.

Table S1. Complexation shift ($\Delta\delta = \delta_{obs} - \delta_{free}$, ppm) of *para*- and *ortho*-proton of DNB moiety and of α-CH proton of both enantiomers of **1**-**7** (30 mM), **8** and **9** (15 mM) in the presence of 1 equiv of (*R*,*R*)-**BTDA**, 1 equiv (**1**-**7**) or 2 equivs of DABCO (**8**,**9**).

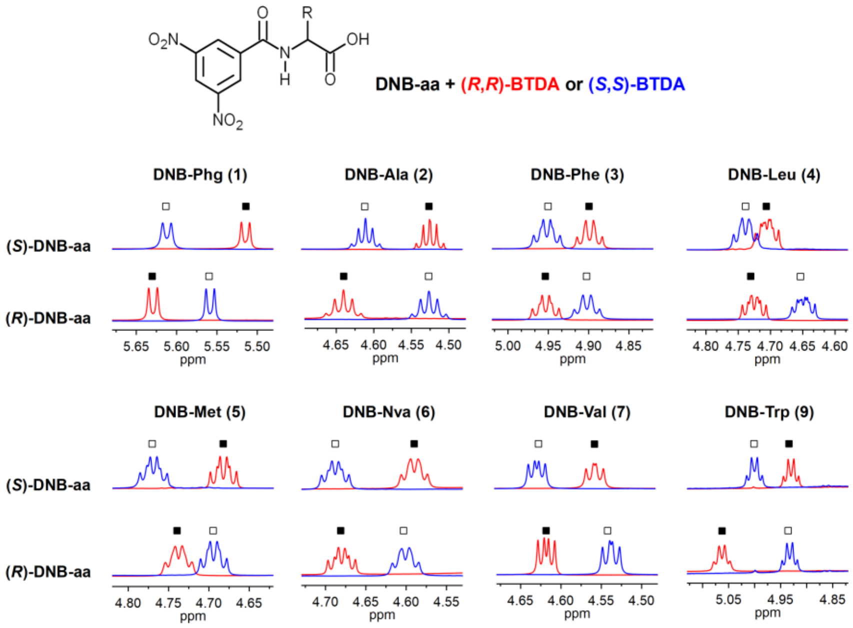

Figure S1. ^{1}H NMR (600 MHz, $CDCl_3$, 298 K) spectral regions corresponding to chiral methine proton of enantiopure **1**-**7** (30 mM) and **9** (15 mM) in the presence of (*R*,*R*)-**BTDA** (red spectra and full symbols) or (*S*,*S*)-**BTDA** (blue spectra and empty symbols), and DABCO.

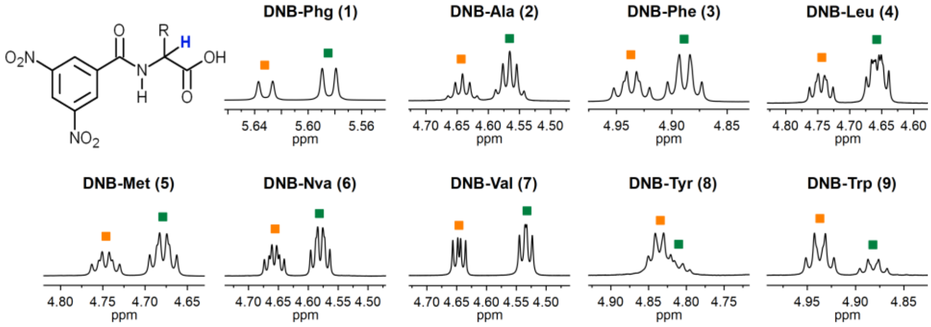

Figure S2. ^{1}H NMR (600 MHz, $CDCl_3$, 298 K) spectral regions corresponding to the methine proton of (*S*)-enantiomerically enriched samples of **1**-**7** (30 mM) in the presence of 1 equiv of (*R*,*R*)-**BTDA** and DABCO and of (*R*)-enantiomerically enriched samples of **8** and **9** (15 mM) in the presence of 1 equiv of (*R*,*R*)-BTDA and 2 equivs of DABCO. Green symbols refer to the (*S*)-enantiomer, orange symbols refer to the (*R*)-enantiomer.

(*R*)-DNB-Ala

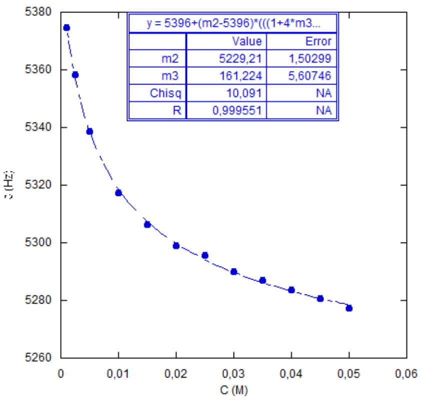

(*S*)-DNB-Ala

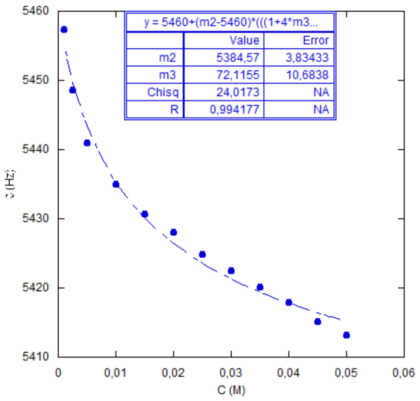

(*R*)-DNB-Phe

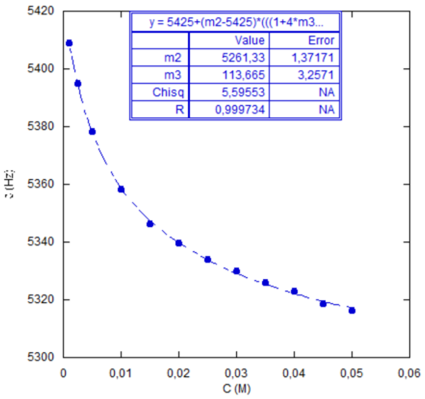

(*S*)-DNB-Phe

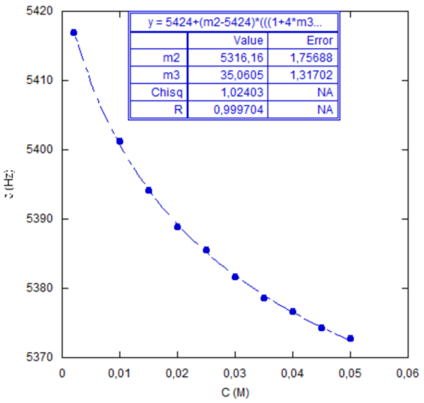

(*R*)-DNB-Leu

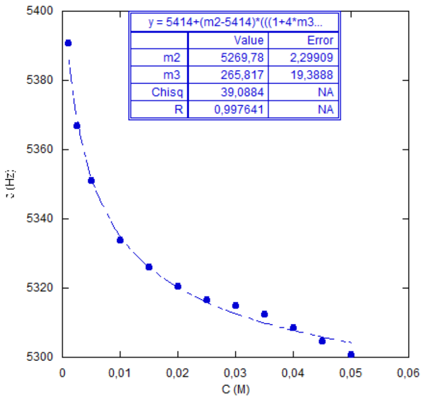

(*S*)-DNB-Leu

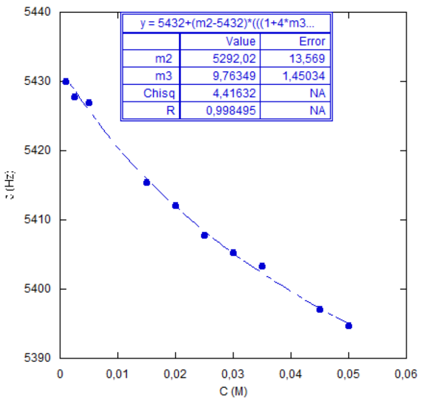

(*R*)-DNB-Met

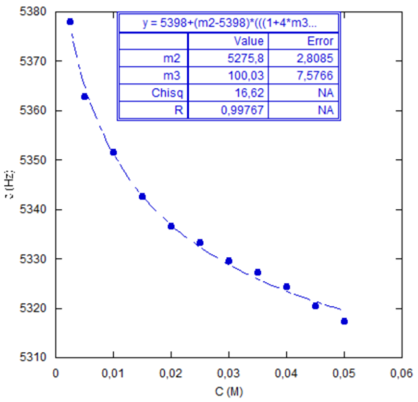

(*S*)-DNB-Met

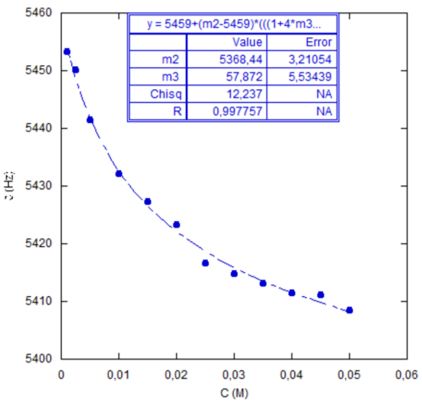

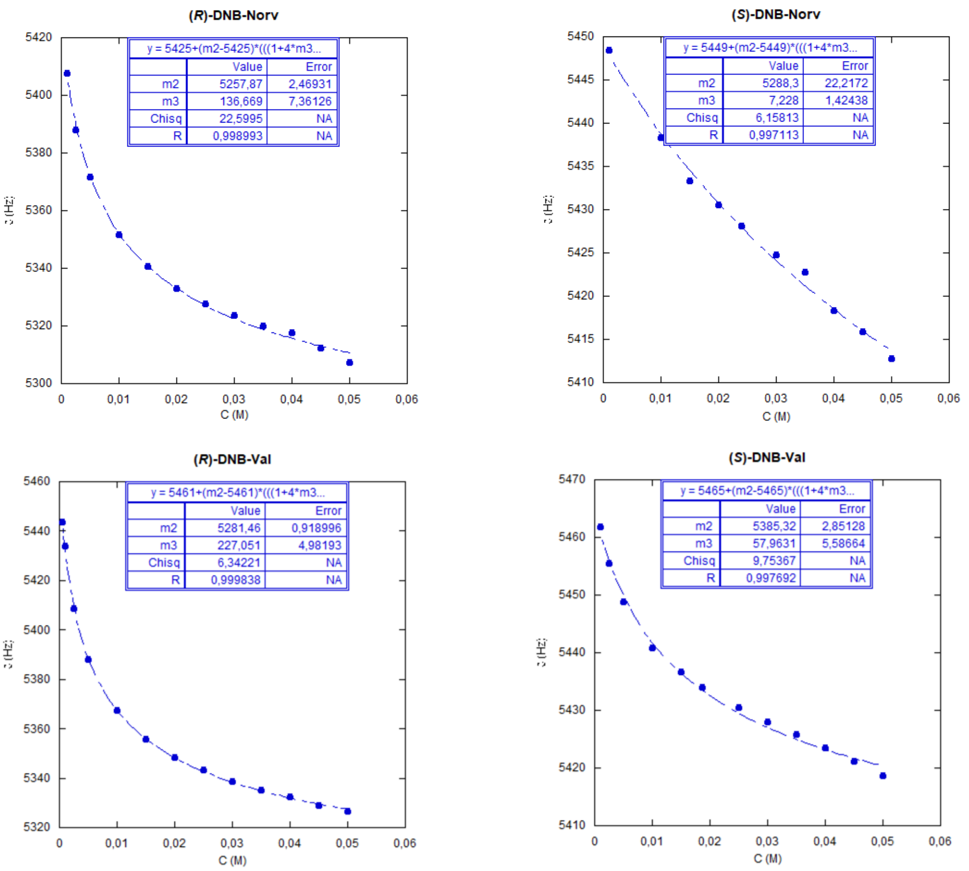

Figure S3. Association constants calculated for the two enantiomers of substrates **2**-**7** in mixture with (*R*,*R*)-**BTDA** and DABCO.

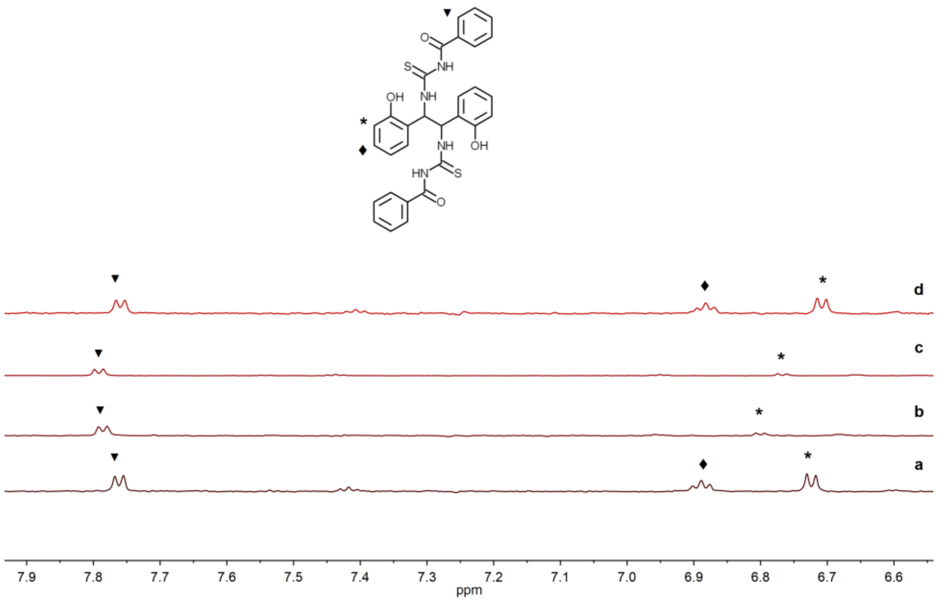

Figure S4. 1D-ROESY (600 MHz, $CDCl_3$, 298 K, mixing time 500 ms) spectra of *ortho*-protons of **2** in (a) (*R*,*R*)-**BTDA**/(*R*)-**2**, (b) (*S*,*S*)-**BTDA**/(*R*)-**2**, (c) (*R*,*R*)-**BTDA**/(*S*)-**2**, (d) (*S*,*S*)-**BTDA**/(*S*)-**2**.

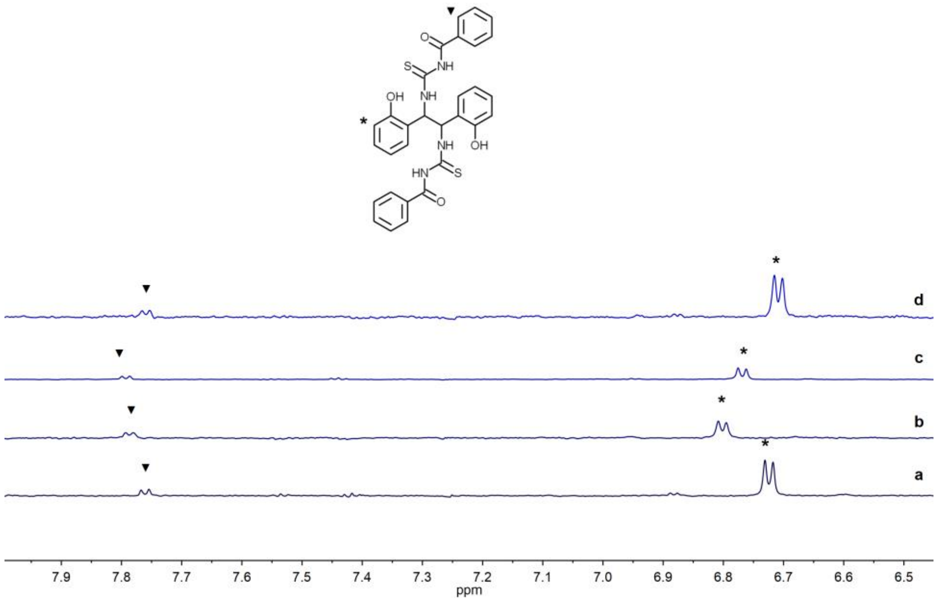

Figure S5. 1D-ROESY (600 MHz, $CDCl_3$, 298 K, mixing time 500 ms) spectra of chiral methine proton of **2** in (a) (*R*,*R*)-**BTDA**/(*R*)-**2**, (b) (*S*,*S*)-**BTDA**/(*R*)-**2**, (c) (*R*,*R*)-**BTDA**/(*S*)-**2**, (d) (*S*,*S*)-**BTDA**/(*S*)-**2**.

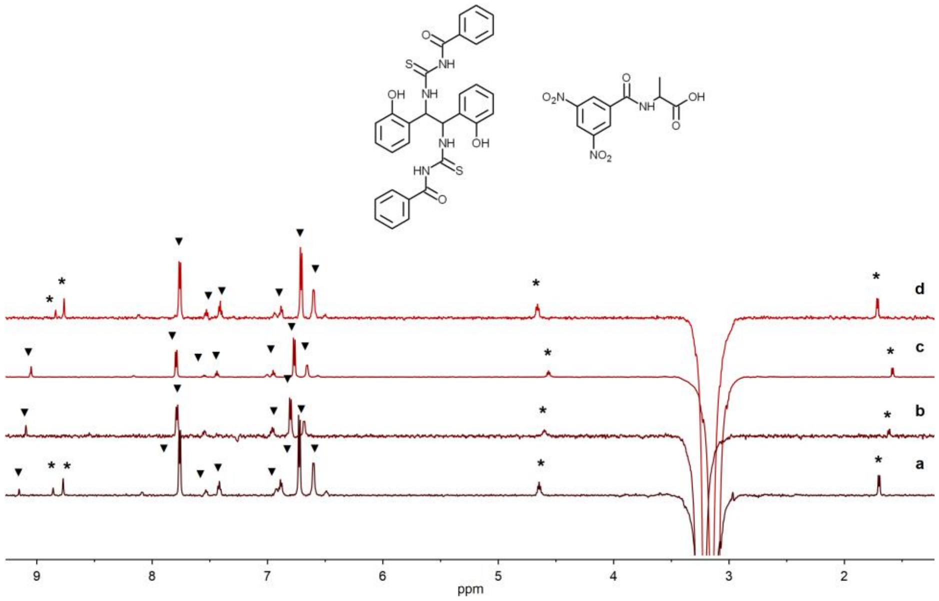

Figure S6. 1D-ROESY (600 MHz, $CDCl_3$, 298 K, mixing time 500 ms) spectra of methylene protons of DABCO in (a) (*R*,*R*)-**BTDA**/(*R*)-**2**, (b) (*S*,*S*)-**BTDA**/(*R*)-**2**, (c) (*R*,*R*)-**BTDA**/(*S*)-**2**, (d) (*S*,*S*)-**BTDA**/(*S*)-**2**. ▼ refers to CSA proton signals, * refers to substrate proton signals.

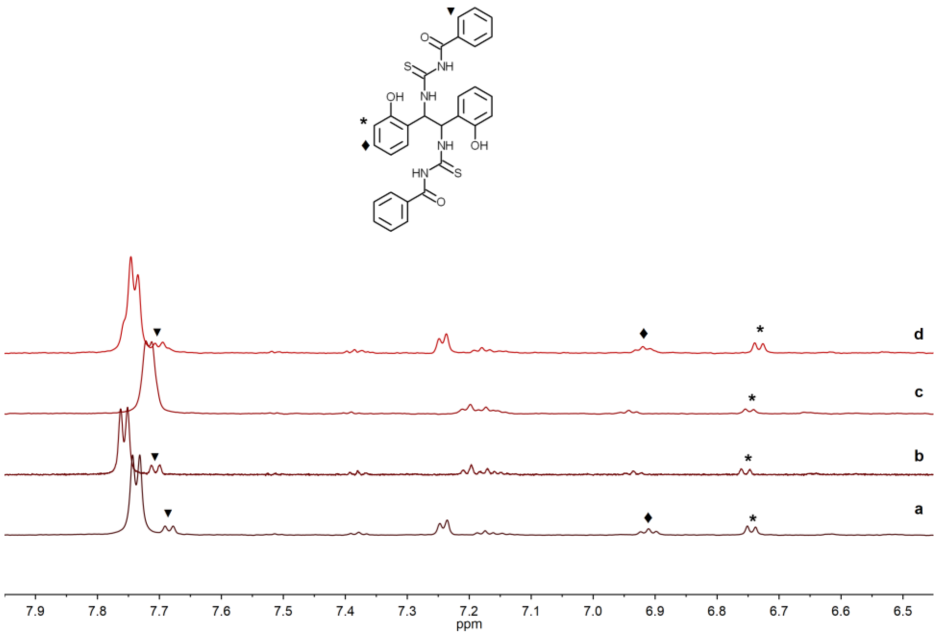

Figure S7. 1D-ROESY (600 MHz, $CDCl_3$, 298 K, mixing time 500 ms) spectra of *ortho*-protons of **3** in (a) (*R*,*R*)-**BTDA**/(*R*)-**3**, (b) (*S*,*S*)-**BTDA**/(*R*)-**3**, (c) (*R*,*R*)-**BTDA**/(*S*)-**3**, (d) (*S*,*S*)-**BTDA**/(*S*)-**3**. Signals without symbols belong to the substrate.

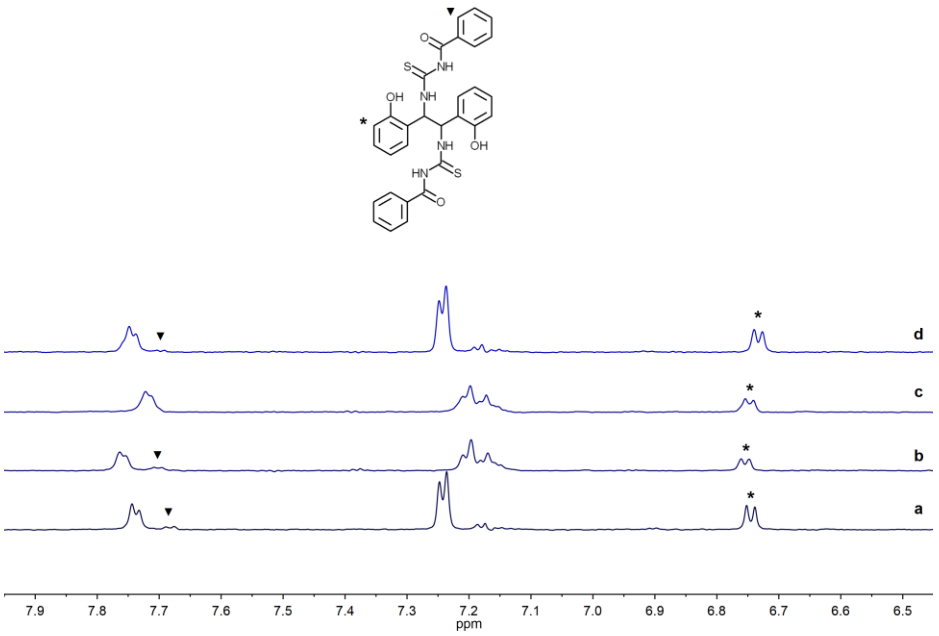

Figure S8. 1D-ROESY (600 MHz, $CDCl_3$, 298 K, mixing time 500 ms) spectra of chiral methine proton of **3** in (a) (*R*,*R*)-**BTDA**/(*R*)-**3**, (b) (*S*,*S*)-**BTDA**/(*R*)-**3**, (c) (*R*,*R*)-**BTDA**/(*S*)-**3**, (d) (*S*,*S*)-**BTDA**/(*S*)-**3**. Signals without symbols belong to the substrate.

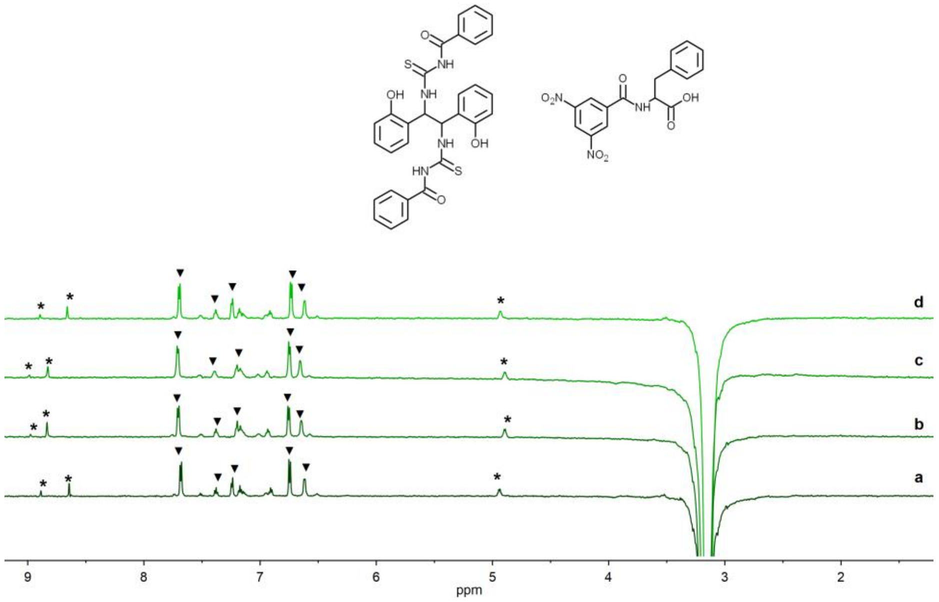

Figure S9. 1D-ROESY (600 MHz, CDCl3, 298 K, mixing time 500 ms) spectra of methylene protons of DABCO in (a) (*R*,*R*)-**BTDA**/(*R*)-**3**, (b) (*S*,*S*)-**BTDA**/(*R*)-**3**, (c) (*R*,*R*)-**BTDA**/(*S*)-**3**, (d) (*S*,*S*)-**BTDA**/(*S*)-**3**. ▼ refers to CSA proton signals, * refers to substrate proton signals.

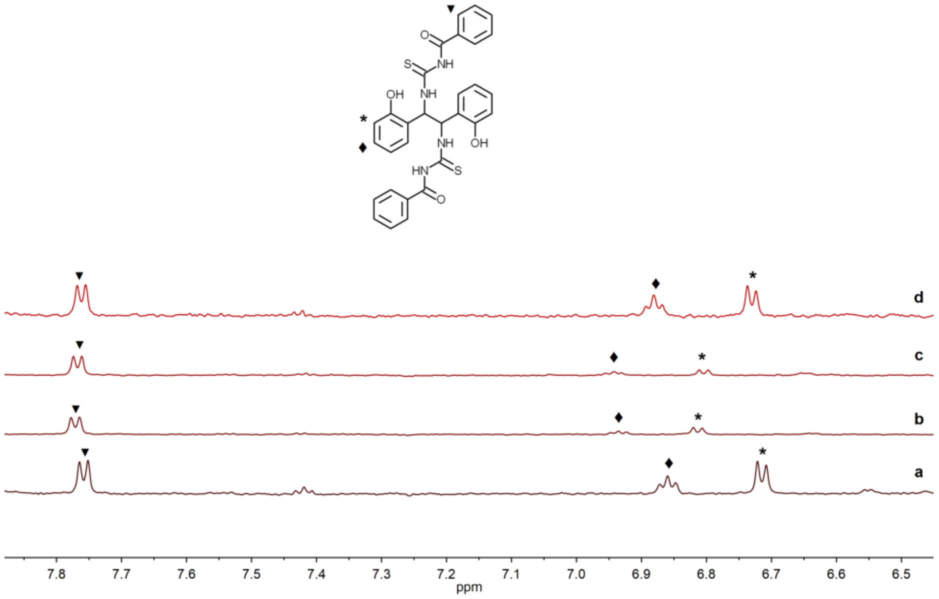

Figure S10. 1D-ROESY (600 MHz, CDCl3, 298 K, mixing time 500 ms) spectra of *ortho*-protons of **4** in (a) (*R*,*R*)-**BTDA**/(*R*)-**4**, (b) (*S*,*S*)-**BTDA**/(*R*)-**4**, (c) (*R*,*R*)-**BTDA**/(*S*)-**4**, (d) (*S*,*S*)-**BTDA**/(*S*)-**4**.

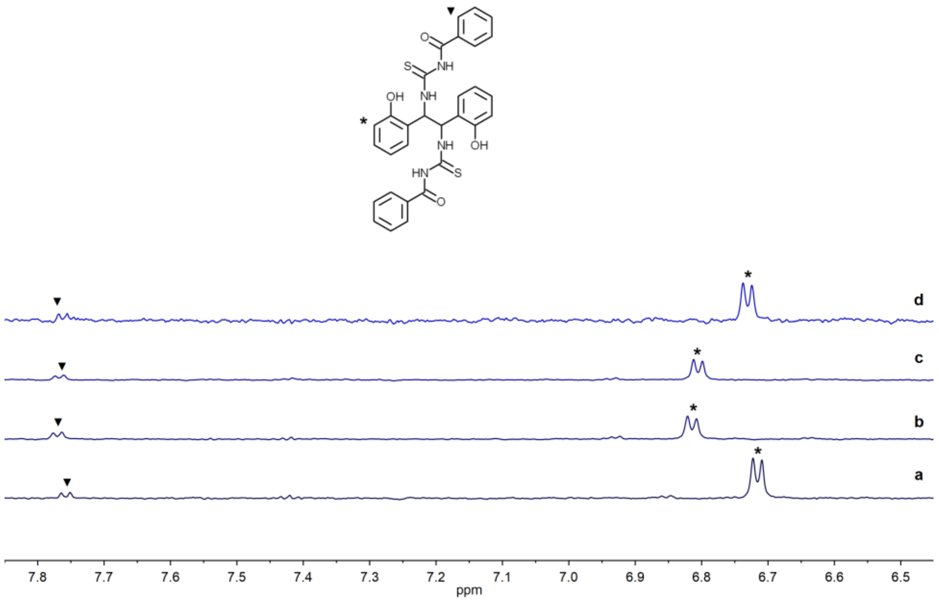

Figure S11. 1D-ROESY (600 MHz, $CDCl_3$, 298 K, mixing time 500 ms) spectra of chiral methine proton of **4** in (a) (*R*,*R*)-**BTDA**/(*R*)-**4**, (b) (*S*,*S*)-**BTDA**/(*R*)-**4**, (c) (*R*,*R*)-**BTDA**/(*S*)-**4**, (d) (*S*,*S*)-**BTDA**/(*S*)-**4**.

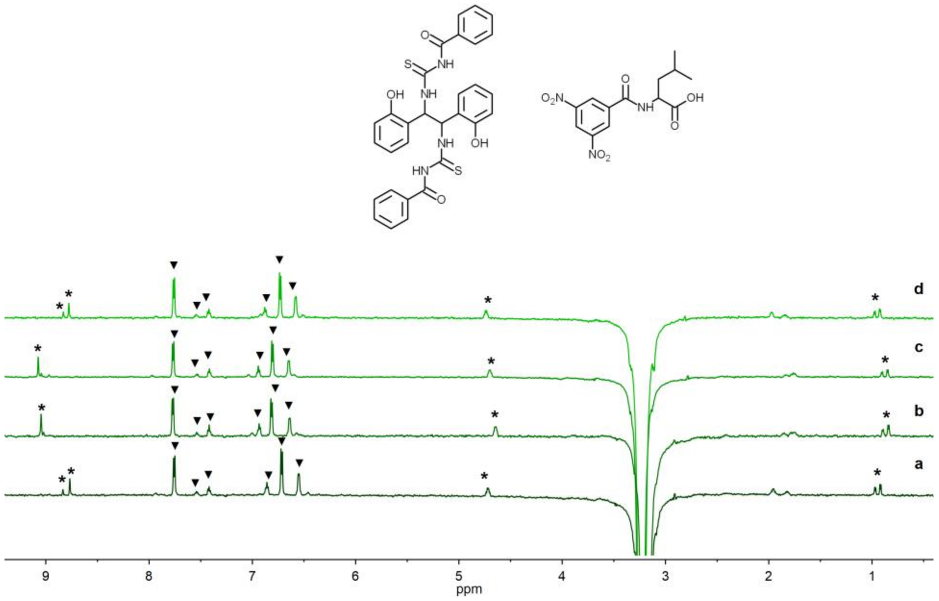

Figure S12. 1D-ROESY (600 MHz, $CDCl_3$, 298 K, mixing time 500 ms) spectra of methylene protons of DABCO in (a) (*R*,*R*)-**BTDA**/(*R*)-**4**, (b) (*S*,*S*)-**BTDA**/(*R*)-**4**, (c) (*R*,*R*)-**BTDA**/(*S*)-**4**, (d) (*S*,*S*)-**BTDA**/(*S*)-**4**. ▼ refers to CSA proton signals, * refers to substrate proton signals.

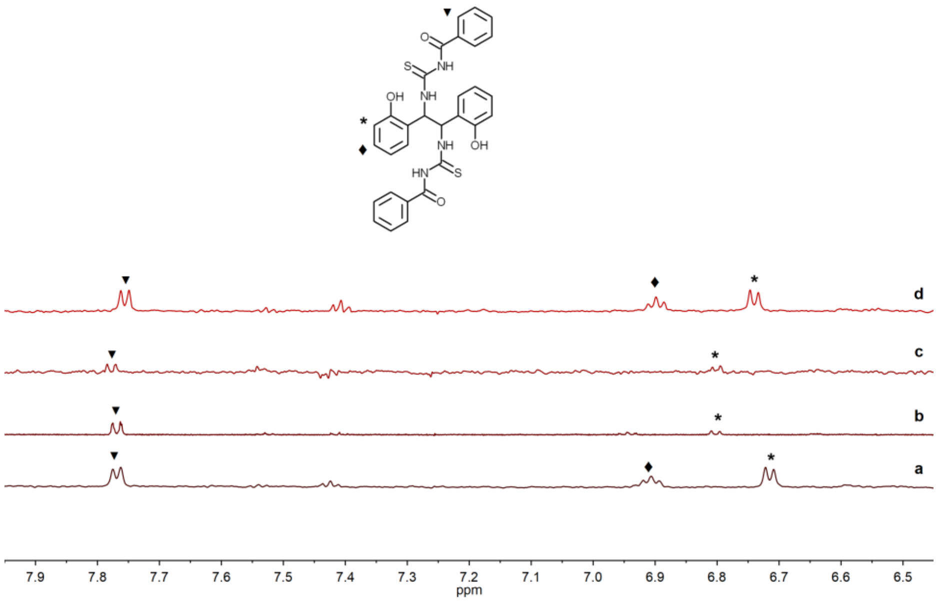

Figure S13. 1D-ROESY (600 MHz, $CDCl_3$, 298 K, mixing time 500 ms) spectra of *ortho*-protons of **5** in (a) (*R*,*R*)-**BTDA**/(*R*)-**5**, (b) (*S*,*S*)-**BTDA**/(*R*)-**5**, (c) (*R*,*R*)-**BTDA**/(*S*)-**5**, (d) (*S*,*S*)-**BTDA**/(*S*)-**5**.

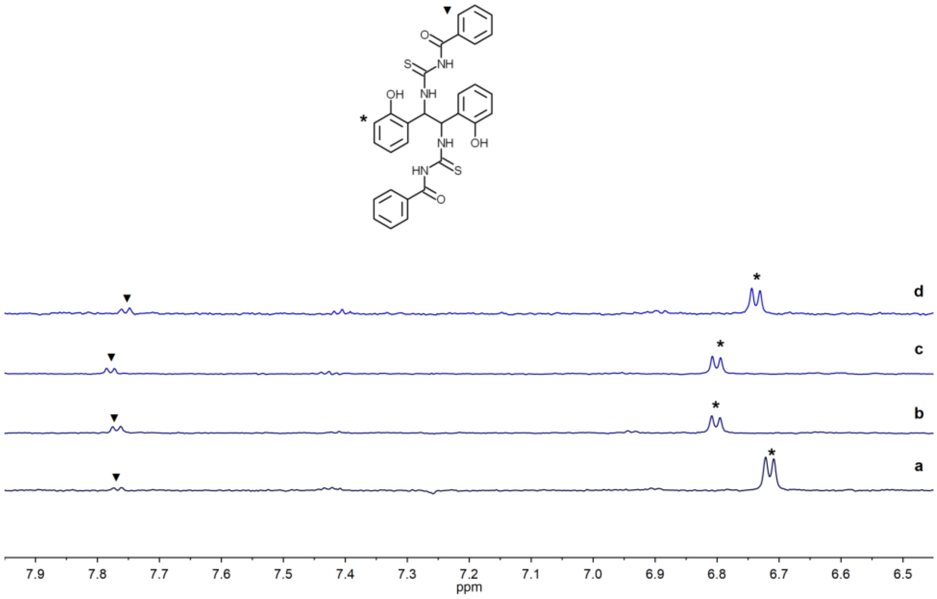

Figure S14. 1D-ROESY (600 MHz, $CDCl_3$, 298 K, mixing time 500 ms) spectra of chiral methine proton of **5** in (a) (*R*,*R*)-**BTDA**/(*R*)-**5**, (b) (*S*,*S*)-**BTDA**/(*R*)-**5**, (c) (*R*,*R*)-**BTDA**/(*S*)-**5**, (d) (*S*,*S*)-**BTDA**/(*S*)-**5**.

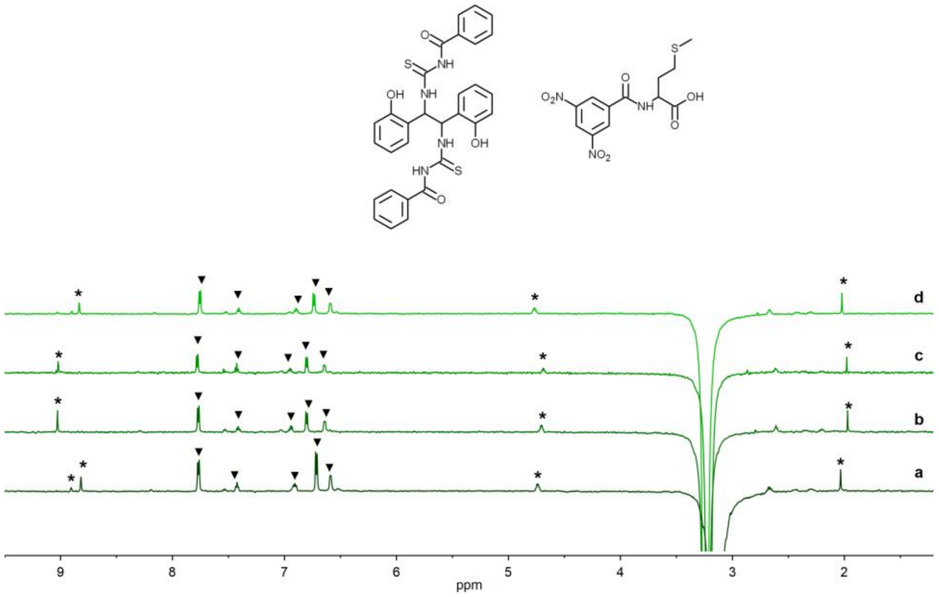

Figure S15. 1D-ROESY (600 MHz, $CDCl_3$, 298 K, mixing time 500 ms) spectra of methylene protons of DABCO in (a) (*R*,*R*)-**BTDA**/(*R*)-**5**, (b) (*S*,*S*)-**BTDA**/(*R*)-**5**, (c) (*R*,*R*)-**BTDA**/(*S*)-**5**, (d) (*S*,*S*)-**BTDA**/(*S*)-**5**. ▼ refers to CSA proton signals, * refers to substrate proton signals.

Table S1. Complexation shift ($\Delta\delta = \delta_{obs} - \delta_{free}$, ppm) of *para*- and *ortho*-proton of DNB moiety and of α-CH proton of both enantiomers of **1**-**7** (30 mM), **8** and **9** (15 mM) in the presence of 1 equiv of (*R*,*R*)-**BTDA**, 1 equiv (**1**-**7**) or 2 equivs of DABCO (**8**,**9**).

Substrate	Δδ (*p*-DNB)		Δδ (*o*-DNB)		Δδ (α-CH)	
	***R*-enantiomer**	***S*-enantiomer**	***R*-enantiomer**	***S*-enantiomer**	***R*-enantiomer**	***S*-enantiomer**
**1**	-0.273	-0.016	-0.252	-0.009	0.158	0.044
**2**	-0.229	-0.049	-0.194	0.064	0.160	0.079
**3**	-0.196	-0.093	-0.218	-0.029	0.161	0.106
**4**	-0.250	-0.046	-0.208	0.092	0.092	0.073
**5**	-0.205	-0.075	-0.205	-0.006	0.012	-0.046
**6**	-0.256	-0.079	-0.245	0.010	0.013	-0.076
**7**	-0.322	-0.109	-0.068	0.053	0.078	0.016
**8**	-0.128	n.d.[a]	-0.127	n.d.[a]	0.087	n.d.[a]
**9**	-0.204	-0.070	-0.119	-0.039	0.215	0.083

[a] not determined due to the unavailability of enantiopure (*S*)-derivative.
